# Turning point: A new global COVID‐19 wave or a signal of the beginning of the end of the global COVID‐19 pandemic?

**DOI:** 10.1002/iid3.606

**Published:** 2022-03-29

**Authors:** Kaixi Ding, Wei Jiang, Chunping Xiong, Ming Lei

**Affiliations:** ^1^ Hospital of Chengdu University of Traditional Chinese Medicine Chengdu China

**Keywords:** breakthrough infections, COVID‐19, mild cases, Omicron variant, SARS‐CoV‐2, vaccine boosters

## Abstract

A new variant named Omicron (B.1.1.529), first identified in South Africa, has become of considerable interest to the World Health Organization. This variant differs from the other known major variants, as it carries a large number of unusual mutations, particularly in the spinous process protein and receptor binding domains. Some specific mutation sites make it vaccine resistant, highly infectious, and highly pathogenic. The world fears that the Omicron variant could be even more harmful than the previous major variant, given that it has emerged amid fierce competition to trigger a new global pandemic peak as infections in South Africa rise. However, some epidemiological evidence has emerged that the Omicron variant may produce milder patient symptoms. We speculate if the virulence of the Omicron variant will diminish as transmissibility increases, thereby signaling the beginning of the end for the global COVID‐19 pandemic. Based on this view, we make recommendations for COVID‐19 mitigation in the present and future. However, it will take a few weeks to determine the true threat posed by the Omicron variant and we need to be fully prepared for future outbreaks, regardless of their severity.

## Graphical abstract

The Omicron variant is highly infectious and highly pathogenic, and some specific mutation sites mean that this variant may be resistant to the vaccine. Some epidemiological evidence has emerged showing that the Omicron variant may lead to milder symptoms in patients. Current vaccines may provide high protection against severe diseases caused by Omicron; therefore, to try to avoid all variants, vaccination and booster shots continue to be recommended.

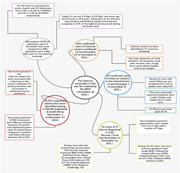

## 1 INTRODUCTION

In early November 2021, a new severe acute respiratory syndrome coronavirus 2 (SARS COV‐2) variant was identified in South Africa and Botswana. On November 26, the WHO renamed the new SARS‐CoV‐2 variant Omicron, after originally naming it B.1.1.529.^1^ It is the fifth “major variant” following α, β, γ, and δ major variants.^2^ The new variant quickly spread to almost all South African provinces, particularly Gauteng. Since the initial discovery of Omicron in South Africa, Australia, Italy, Germany, the Netherlands, Israel, Hong Kong, the United Kingdom, Botswana, and Belgium have all reported Omicron cases.^3^ Compared with the SARS‐CoV‐2 (Wuhan‐Hu‐1, GenBank accession number NC_045512.2) reference strain, the variant consensus sequence contains 44 amino acid substitutions, 6 amino acid deletions, and 1 amino acid insertion.^4^ Compared with the original SARS‐COV‐2 strain, there were at least 30 amino acid substitutions, 3 small deletions, and 1 tiny insertion in the spike glycoprotein gene, and 15 of the mutations (residues 319−541) were in the receptor‐binding domain (RBD).^5^, ^6^ Among these genetic variations, spike mutations make detecting potential Omicron cases more difficult, and mutations in the S protein RBD, caused by this novel variant, potentially affect infectivity and antibody resistance.^7^

Due to the uncertainty about how these detected mutations affect the efficacy of existing vaccines, and the transmissibility of the Omicron variant, several countries have reacted quickly to the potential Omicron variant prevalence. Some countries in Asia and Europe have imposed emergency travel bans and restrictions on South Africa and its neighbors.^2^ The world is worried about whether the Omicron mutation could lead to another severe global pandemic. However, researchers in the United States and South Africa have recently discovered that most Omicron cases are mild.^8^, ^9^, ^10^ Although the Omicron variant can still be transmitted among vaccinated people, it may not cause serious illness due to its high infectivity, and changes could cause it to attenuate itself, just as the Spanish flu did.^11^, ^12^ Some scholars have proposed that the special security measures to prevent and control SARS‐CoV‐2 infection will end after the massive infection wave triggered by the Omicron variant. This is based on the wide distribution of earlier virus variants, immunization with newer vaccines, availability of improved antiviral drugs, and existing protection for vulnerable populations in future waves. However, it may continue to exist as recurrent disease.^13^ We continue to face threats and challenges posed by other new variants and unknown superviruses.^14^ As such, we must actively develop appropriate and continuously optimized responses for the current and rapidly evolving fifth wave of the Omicron pandemic. On a positive note, we wonder is it possible that a new strain may “signal the end of COVID‐19.” However, accurate conclusions about the true threat of Omicron will take weeks to obtain. Meanwhile, we all need to be prepared for a potential battle. In this review, we summarize two different global predictions for the future of the Omicron variant and make recommendations for the prevention and control of the new Omicron pandemic wave.

### 1.1 Omicron is not a good comer

**Figure 1:**
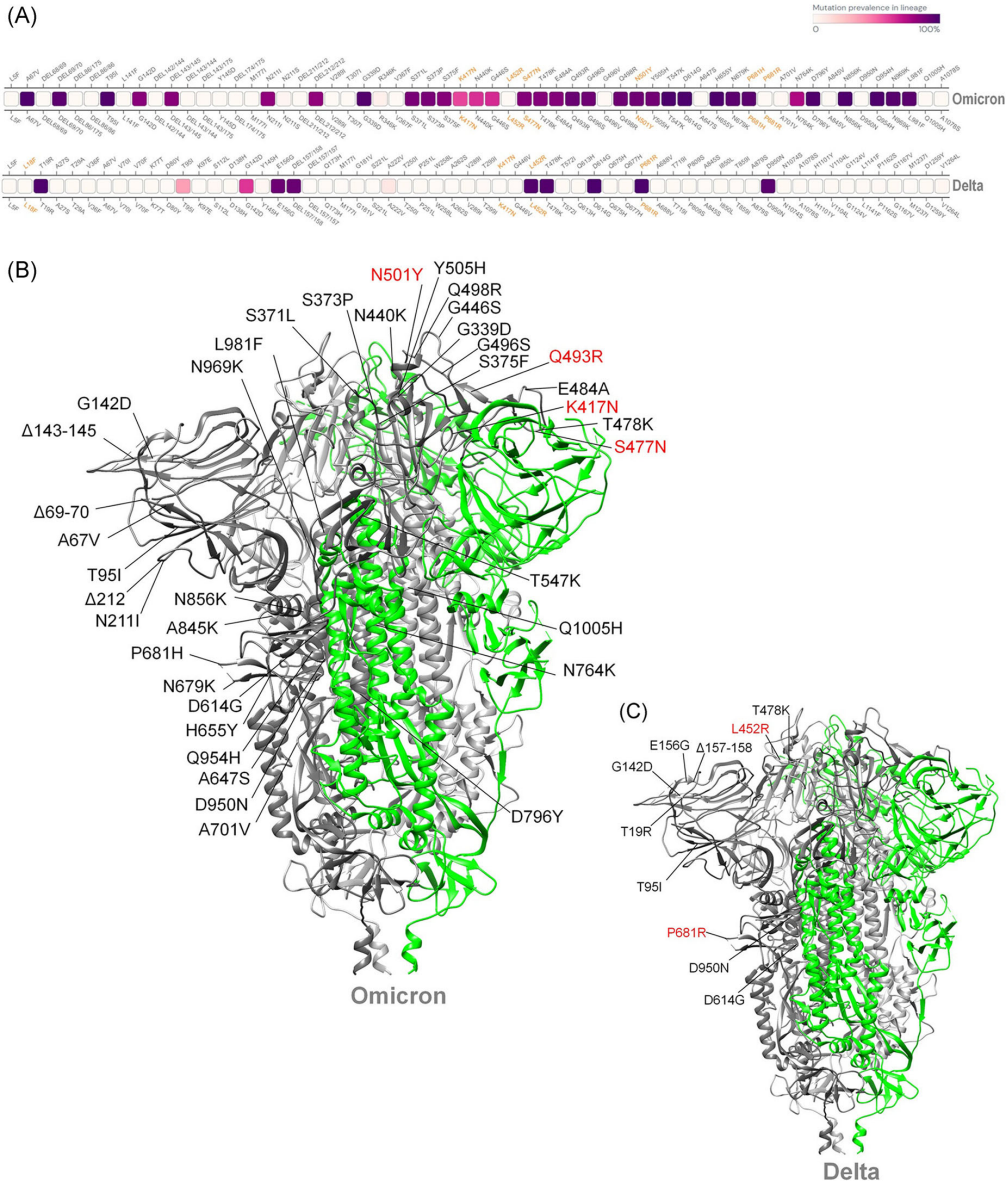
Mutation hotspot of Omicron SARS‐CoV‐2 spike glycoprotein. (A) Comparative mutational hotspots of spike glycoprotein of Omicron and Delta variant of SARS‐CoV‐2 (the gradient of the dark purple coding box represents the prevalence of the mutation throughout the sequenced samples). (B) The structure of spike glycoprotein of Omicron SARS‐COV‐2 shows comparative mutation. (C) The structures of spike glycoprotein of Delta SARS‐CoV‐2 show the comparative mutations^6^

The new SARS‐COV‐2 mutant (Omicron) basic infectivity and antibody resistance are determined by a mutation in the Spike (S) protein RBD. Omicron is highly mutated compared with other major VOCs such as Delta, classified b.1.617.2, and this may be associated with higher transmissibility, stronger virus binding affinity, and antibody resistance (Figure 1). Based on an experimentally proven deep learning model, Chen et al. revealed that Omicron is roughly 10 times more infectious than the original virus, and twice as infectious as the Delta variant. Furthermore, Omicron's vaccine‐evasion capability is about twice as high as that of the Delta variant.^15^ Moreover, due to its triple mutation at the furin‐cleavage site, such as H655Y, N679K, and P681H, the Omicron variant is easily transmitted. By increasing the type and frequency of the Spike:655Y cleavage, it increases peak protein processing and the potential of promoting fusion, which leads to adaptive mutation.^16^ Such a mutation is also highly likely to make it the resident novel Coronavirus of the future. In late November a surge in South African Omicron cases seemed to confirm the prediction that Omicron is relatively contagious.^17^ Two Omicron patients, fully vaccinated against COVID‐19, have been found in Hong Kong,^18^ which supports the other prediction that the Omicron variant can escape the vaccine. Considering that the double‐mute Delta variant has wreaked havoc on the Indian healthcare system and those of its neighbors,^19^ the highly silent Omicron variant has also raised global concerns that at its peak it may be able to ward off antibodies produced by viruses which have previously been infected against, or coronavirus vaccines.^20^ The virus source remains unknown, and there are currently three hypotheses about the origin of the Omicron variant. The first hypothesis is that the Omicron variant came from a secluded place and evolved. If the true origin of the virus has not yet been discovered, is it true that the virus spread a long time ago? The second hypothesis is that viruses may be dormant in rodents or other animals, rather than humans, and thus undergo different evolutionary pressures to select for new mutations. Considering that a young woman in South Africa with uncontrolled HIV infection carried SARS‐COV‐2 for more than 6 months, a third hypothesis speculates that the virus may have accumulated mutations during chronic infection in immunocompromised populations. If this is true we need to close the gaps in the HIV treatment cascade before the virus accumulates mutations in AIDS patients.^21^ Regardless of the origin of Omicron, or some other future hypotheses, it must be taken seriously, given the perceived dangers.

### 1.2 Could the Omicron variant be the tipping point for the beginning of the end of the COVID‐19 epidemic?

Currently, it is not clear whether Omicron causes more severe disease than other variants, including Delta.^22^ To date, the available epidemiological evidence suggests that Omicron variants cause more mild infections than severe ones.^11^ From November 24 to December 9, 2021, there were 2152 confirmed cases of Omicron variants worldwide. Infection occurred more in young and middle‐aged people than in minors, and cases were generally “mild.” Clinical symptoms include headache, body pain, muscle pain, cough, fever, systemic myalgia, and severe fatigue.^12^ From December 1 to December 8, at least one Omicron variant case was detected in 22 states in the United States, including some cases that indicate community transmission. In the initial follow‐up of 43 cases, 1 case was hospitalized, and no patients died. Of these, 25 (58%) were aged 18−39, and the most commonly experienced symptoms were cough, fatigue, and stuffy, or runny nose.^8^ An epidemiological investigation by Brandal in Norway, from late November to December 13, 2021, discovered that following a large Christmas party in Oslo, Norway, there was an outbreak of Omicron SARS‐COV‐2. In 96% of the Omicron variant infections, the patients were fully vaccinated, and thus far no cases have needed hospitalization. Of the 81 cases diagnosed, the most common symptom was cough (83%), followed by cough, runny/stuffy nose (78%), fatigue/lethargy (74%), sore throat (72%), headache (68%), and fever (54%). Symptom severity was graded from 1 (no symptoms) to 5 (significant symptoms). Of the 79 cases, 49% presented Grade 3 symptoms and, 11% presented Grade 4 symptoms.^23^ This early epidemiological data provide preliminary verification that Omicron variants tend to cause milder infections, and are highly contagious in fully vaccinated young and middle‐aged adults. Recent laboratory studies have found that TMPRSS2 cuts the Omicron spike protein less efficiently than other variants, thus blocking the pathway through which Omicron virus particles can directly enter cells. Furthermore, since TMPRSS2 is more common in lower airway cells, Omicron may protect the lungs and cause milder disease.^24^

Early observational data in some South African health systems also support the evidence that Omicron infections tend to be less severe. A recent study by Pulliam et al. found that in the city of Tshwane, Gauteng, COVID‐19 patients hospitalized with Omicron infections, between 30 and 80+ years of age, had significantly lower rates of severe illness compared with the same age group patients with the Delta infection. However, the average severity of Omicron cases could of course rise. One possible reason for this not happening is that Omicron mutations cause milder disease. That would partly offset the surge in cases, but the death rate could rise if hospital wards are overwhelmed. Another explanation is that many older South Africans have been vaccinated recently. If this is the source, Omicron would pose a serious threat to unvaccinated people.^25^ In the same period in Tshwane, South Africa, after the early Omicron variant virus infection outbreak, the severity of COVID‐19 disease in large hospitals has declined. According to a recent cross‐sectional survey by Abdullah et al., 466 inpatients have died from COVID‐19 since November 14, 2021, which is a lower rate than the 3962 COVID‐19 patient deaths since May 2020. The peak bed occupancy rate during the Omicron wave was 51% of the Delta wave peak. The mortality rates from the Omicron and Delta waves were 4.5% and 21.3% (*p* < .00001), respectively. The ICU admission rate during the Omicron and Delta waves was 1% and 4.3% (*p* < .00001), respectively. The average illness length of the Omicron and Delta waves was 4.0 and 8.8 days, respectively, and the mean age of Omicron and Delta patients was 39 and 49.8 years, respectively.^9^

**Figure 2:**
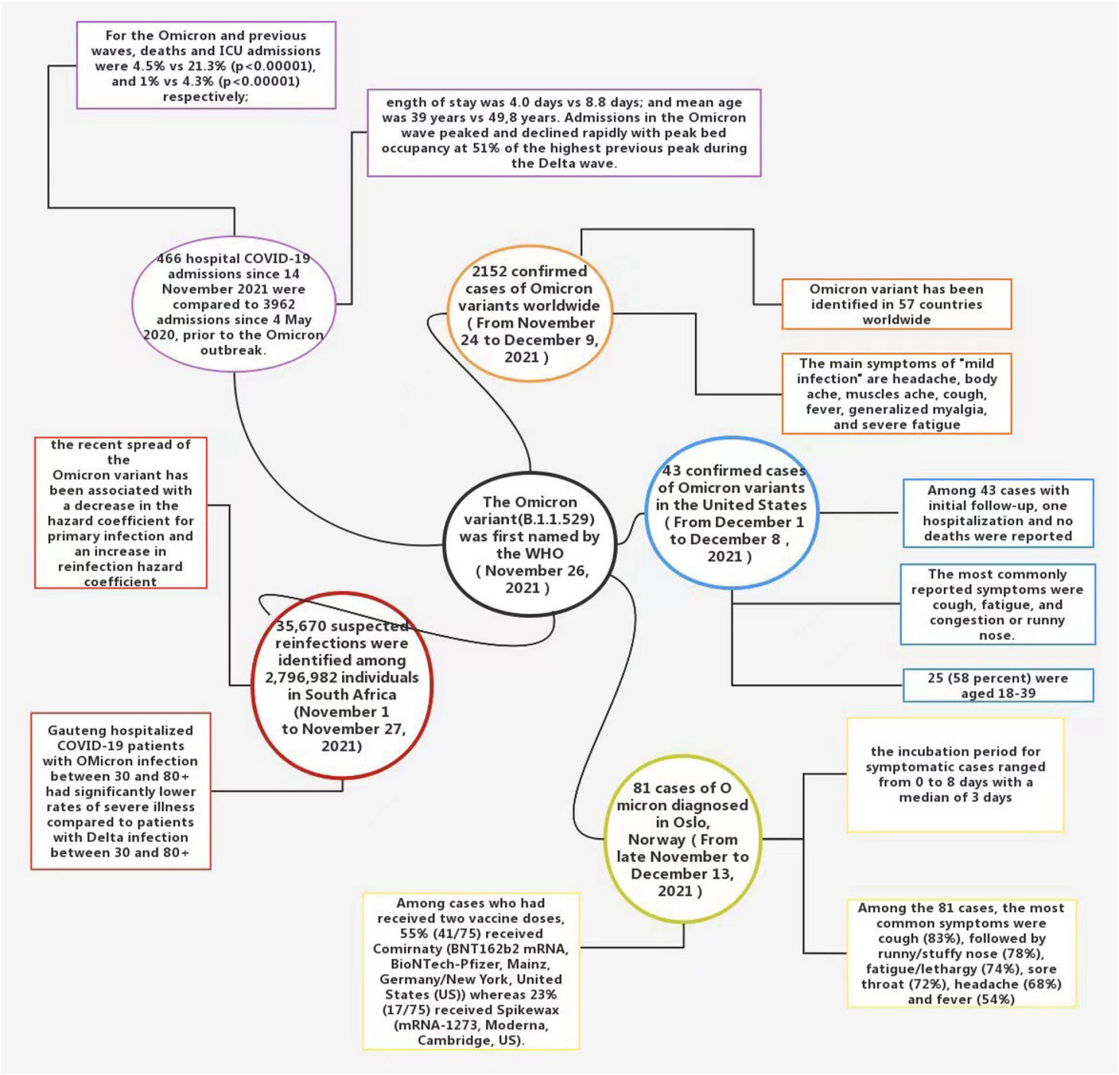
Early epidemiological evidence supports milder symptoms of Omicron variant infections^8^, ^9^, ^12^, ^23^, ^25^

These recent epidemiological reports indicate that the Omicron variant is highly infectious to young and middle‐aged people with strong resistance and appears more in the form of the mild disease (Figure 2). If this pattern of “low virulence and high transmission” continues globally, we are likely to eventually see a complete decoupling of infections and deaths, suggesting that Omicron could be a harbinger of the end of the COVID‐19 epidemic. However, there are still too few Omicron variant infection cases to draw meaningful conclusions about whether the variant causes milder disease.

### 1.3 Acquired immunity to Omicron infection may contribute to the end of the COVID‐19 pandemic

Recently, at the 150th session of the Executive Board (January 24, 2022), WHO director‐general Tedros Adhanom Ghebreyesus stated that, “There are different scenarios for how the pandemic could play out and how the acute phase could end. But it is dangerous to assume that Omicron will be the last variant or that we are in the endgame.”^26^ There are still a number of other novel coronavirus variants that continue to emerge such as IHU (B.1.640.2) with 46 mutations. In addition, there are also combinations of two existing VOCs, such as Delmicron (a combination of Omicron and the most lethal variant Delta) which have yet to be scientifically confirmed.^14^ The constant mutation of this virus and the unknown potential of new virus properties seem to make the new Omicron pandemic wave unpredictable.

However, at this stage, while continuing to focus on new novel coronavirus variants, we should focus on the existing Omicron variant itself. Omicron variants are now more transmissible than the Delta, Alpha, Beta, and Gamma variants, and some clinically used antibodies may fail to respond to Omicron variants.^13^, ^27^, ^28^ However, on the positive side, the acquired immunity generated by an Omicron infection itself seems to have contributed favorably to the end of the pandemic.

A recent cross‐sectional epidemiological study of 10 breakthrough Omicron cases vaccinated with Pfizer's BNT162b2 or Johnson & Johnson's AD26.COV2.S vaccine was conducted. In this study, the neutralization rate of the Delta virus increased from 129 to 790 (6.1‐fold) in vaccinated participants. However, it only increased from 18 to 46 (2.5‐fold, not statistically significant) in unvaccinated participants.^29^ Another study by Keeton et al. found that the number of Omicron cross‐reactive T cells was similar to Beta and Delta variants as well as in hospitalized patients infected with Omicron (*n* = 19). The response of T cells to ancestral spindles, nucleocapsid, and membrane proteins is comparable with that previously observed in hospitalization waves dominated by the ancestral, Beta or Delta variants (*n* = 49).^30^ A study by Ahmed et al. screened all S‐Specific 224 CD8+ and 167 CD4+ SARS‐COV‐2 T‐cell epitopes provided by The Immune Epitope Database (IEDB). It was found that for Omicron‐defining mutations, 14% of CD8+ and 28% of CD4+ T‐cell epitopes contain at least one Omicron mutation. This indicates that the vast majority of CD8+ and CD4+ T‐cell epitopes (86% and 72% respectively) are unaffected by Omicron.^31^ These results suggest that Omicron infection and postvaccination Omicron infection may enhance immune protection against Omicron and other variants. This protective effect may be mainly related to T cells assisting in B‐cell activation to produce antibodies and helping to provide protection against disease by eliminating virus‐infected cells.^31^ In addition, an experiment using NTD‐specific probes to focus on NTD‐resistant memory B cells in a group of pre‐Omicron‐infected individuals was conducted by Wang et al. It was found that SARS‐COV‐2 infection and/or Wuhan HU‐1 mRNA vaccination produced a variety of memory B cells. These produced anti‐NTD antibodies, among which some neutralized the associated mutations.^32^ These associated memory B cells are able to rapidly deploy to the antibody‐secreting plasma cell compartment after reinfection. This may contribute to the relatively benign process of subsequent infection with SARS‐CoV‐2 variants including Omicron.

An Omicron infection tends to be mild and there is evidence from the above clinical studies suggesting that Omicron infection provides acquired immune protection. As such, it does not seem that an Omicron epidemic would be a bad thing for improving overall global immunity to COVID‐19. However, the Omicron pandemic was still a catastrophic blow for vulnerable groups and global containment efforts. Therefore, active immunization provided by existing vaccines and broadly neutralizing mAbs‐recognizing RBD epitopes conserved across SARS‐CoV‐2 variants are indispensable for controlling the ongoing Omicron pandemic.^33^

### 1.4 Be prepared for both

While the Omicron variant exhibits immune escape characteristics, it may be less virulent than the Delta variant as it becomes more infectious, which could bode well for the overall picture. This suggests that the virus could still spread from person to person but would be less life‐threatening.^34^, ^35^ However, fully understanding the severity of the Omicron variant will take time. All COVID‐19 variants, including the dominant Delta variant, can cause severe illness or death.^22^ No country currently has a health system that can withstand and defend against a major outbreak, even of the potentially less virulent Omicron strain. The research mentioned above confirms that the Omicron variant may be more transmissible, that existing vaccines may be less effective at preventing infection than with Delta, and that SARS‐CoV‐2 Omicron VOC is highly contagious in fully vaccinated young and middle‐aged people.^8^, ^23^ In addition, Omicron is more likely to escape current vaccine‐induced immune protection^36^ than the prototype, or other variants. The results of a preliminary laboratory study published by Pfizer and BioNTech show that individuals who received two doses of the current COVID‐19 vaccine had a more than 25‐fold reduction in neutralizing titer against the Omicron variant.^37^ In a cross‐status survey conducted by Garcia‐Beltran et al., serum samples from 88 mRNA‐1273, 111 BNT162b, and 40 AD26.cov2.s vaccinees were collected. The Omicron neutralizing effect was not detected in the serum of most vaccinators.^38^ An in vitro Omicron trial is soon to be conducted by Delphine Planas. When detecting the sensitivity of the infectious Omicron virus isolated from travelers returning from Egypt, antibodies present in nine monoclonal antibodies (mAbs), and 115 serum samples from COVID‐19 vaccinators or recovered persons, Omicron was found to be completely or partially resistant to neutralization of all mAb‐tested samples. Serum samples from Pfizer or AstraZeneca vaccinees taken 5 months after full vaccination showed little Omicron inhibition.^39^

**Figure 3:**
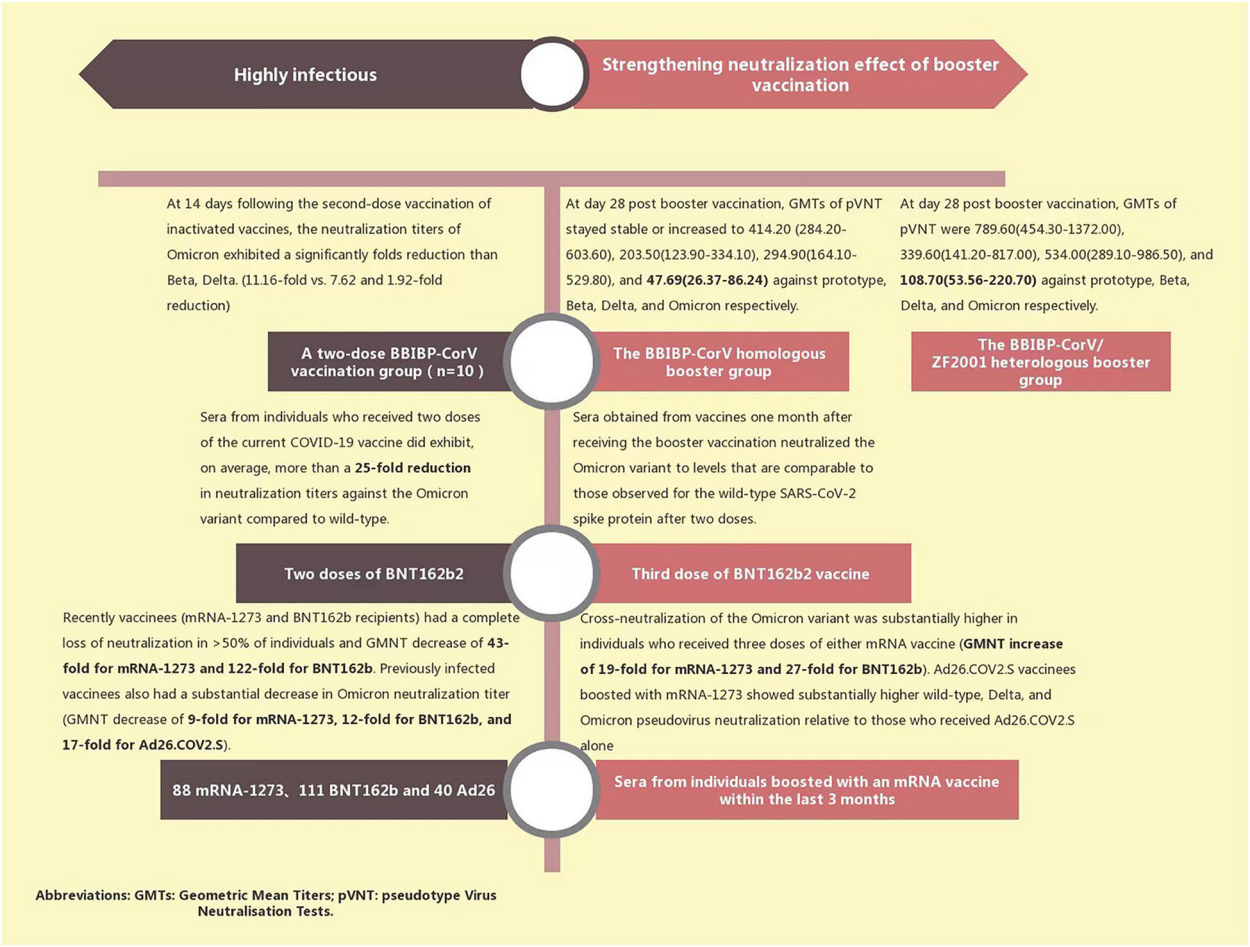
Neutralization of some existing vaccines and boosters against Omicron variants^37^, ^38^, ^39^

Although existing evidence suggests that Omicron mutants can cause vaccine breakthrough infections, a positive note is that Omicron's ability to evade neutralizing antibodies does not mean that vaccination, and immune responses triggered by previous infections, will not protect against the variant.^5^ An in vitro experiment by Ai found that after two doses of inactivated whole virion vaccine injected as “primed,” a third heterologous protein subunit vaccine (BBIBP‐CorV homologous booster) and a vaccine booster of a homologous (BBIBP‐CorV/ZF2001 heterologous booster) inactivated vaccine increased neutralization against Omicron.^36^ Preliminary laboratory studies by Pfizer demonstrate that those who received a third dose, or booster vaccine, neutralized the Omicron variant to levels comparable with the wild‐type SARS‐CoV‐2 spike protein observed after two doses. The third dose strongly increased CD8+ T‐cell levels targeting multiple spike protein epitopes which are thought to be related to protection against severe disease.^37^ Receiving a third dose of an mRNA‐based vaccine effectively produces an effective cross‐neutralization response against SARS‐CoV‐2 Omicron by increasing breadth and cross‐neutralization antibody reactivity.^38^ Some studies propose that heterologous vaccine boosters are more effective than homologous vaccine boosters.^40^ Current vaccines may provide high protection against severe diseases caused by Omicron; therefore, to try to avoid all variants, vaccination and booster shots continue to be recommended^41^ Figure 3). Vaccination and boosters not only reduce the pressure of infection from the existing Delta variant but also buys us time to develop a vaccine and booster for the Omicron variant.^24^ In addition to providing additional neutralization, the combination of vaccine and booster may also reduce the transmission risk for the Omicron variant by speeding up viral clearance and reducing quantitative infectious viral titers.^42^ Subsequently, whether the next Omicron wave turns out good or bad, we will be well prepared.

## 2 RECOMMENDATIONS

Early genetic and clinical data suggest that Omicron variants are immune evasive, highly infectious, reinfective, and prone to milder infections, but confirmation of these data will require large sample sizes and reliable experimental results.^6^, ^15^, ^25^ Healthcare systems around the world are facing the twin challenges of Omicron and Delta, and there is concern that some research has discovered that immunocompromised people may carry both strains. A new double‐spike variant, “Delmicron,” may be both highly virulent and highly infectious. However, this hypothetical “Delmicron” requires further exploratory research and analysis and is effectively certified by the WHO.^43^ Therefore, looking at the current global epidemic, we need to prioritize both the prevailing “Delta” and Omicron variants, the latter of which has a potentially high transmission rate and may become the dominant variant in the future.

The traditional vaccination and behavioral changes (wearing masks and reducing population density and personal contact) epidemic control model in the Olympic and paralympic games in Tokyo, Japan (at the same time as Japan's fifth pandemic wave) still apply to curb the spread of the virus in the world today, facing the Delta and Omicron variants.^44^ Hierarchical management of different risk areas effectively contained the spread of Omicron in South Africa, and with daily vaccination rates increasing 10‐fold in every province of South Africa, the daily overall onset risk was only reduced by 0.34%−7.86%.^45^ The spatiotemporal spread of Omicron was controlled by combining preventive measures and existing vaccines. Existing COVID‐19 prevention measures have proved equally effective for Omicron and should continue to be recommended.^46^

However, existing vaccines are less effective against Omicron's neutralizing antibodies, and it is necessary to further investigate the targeting of conserved immunogenic vaccines, or vaccines with other variants of proteins, which can protect Omicron against current and future growth. The creation of preventative, multivalent, or protein‐based vaccines may confer better protection.^36^, ^37^, ^38^, ^46^ However, preparation of a specific Omicron vaccine is still on schedule and is expected to take at least 3 months (from November 25, 2021).^37^ Additionally, a new universal coronavirus vaccine and booster needle tailored for Omicron are in development.^47^, ^48^ Existing booster injections can enhance Omicron neutralization on the basis of two prior doses.^36^, ^37^ Additionally, the existing vaccine booster jab protects against symptomatic or asymptomatic infection, transmission, and severe infection by promoting specific CD8+ or CD4+ T‐cell response. It also neutralizes memory B‐cell receptor (BCR) width and weak respiratory mucosal neutralizing Ab responses.^42^, ^49^, ^50^, ^51^, ^52^ Given these facts, some countries have been quick to respond. Among them, the United States continues to push the current program of two vaccine doses plus booster shots, which can enhance Omicron neutralization, especially in vulnerable groups such as the elderly, pregnant women, and immunocompromised populations.^53^ In addition to traditional behavioral change measures to attempt to slow the spread of the Omicron variant in the United Kingdom, the UK Vaccine Enhancement Program has extended to the over 40s. Moreover, the original second dose to booster time has been shortened from 6 to 3 months.^54^ In addition, novel vaccine formulations are being developed, such as egg antibodies using the RBD of the SARS‐CoV‐2 spike protein. These include immunoglobulin Y where IgY nose drops capture nasal mucosal viruses and α‐galactoside amide (αGalCer), a potent constant natural killer T‐cell (iNKT) agonist. It binds to protein antigens as an adjuvant to induce significantly stronger humoral and cellular responses. A comprehensive evaluation was conducted on the extensive protective effect of ACE2‐FC combined neutralizing antibodies against SARS‐CoV‐2 and its variants.^55^, ^56^, ^57^ Until specific Omicron vaccines are available, new universal vaccines and booster shots tailored to Omicron have been successful. Consequently, perhaps this two‐dose vaccine plus the current booster is the best option for vaccine treatments. Vaccines and booster shots should be allocated according to priority categories, particularly among vulnerable populations with low immunity, or health workers in urgent need.^58^

**Figure 4:**
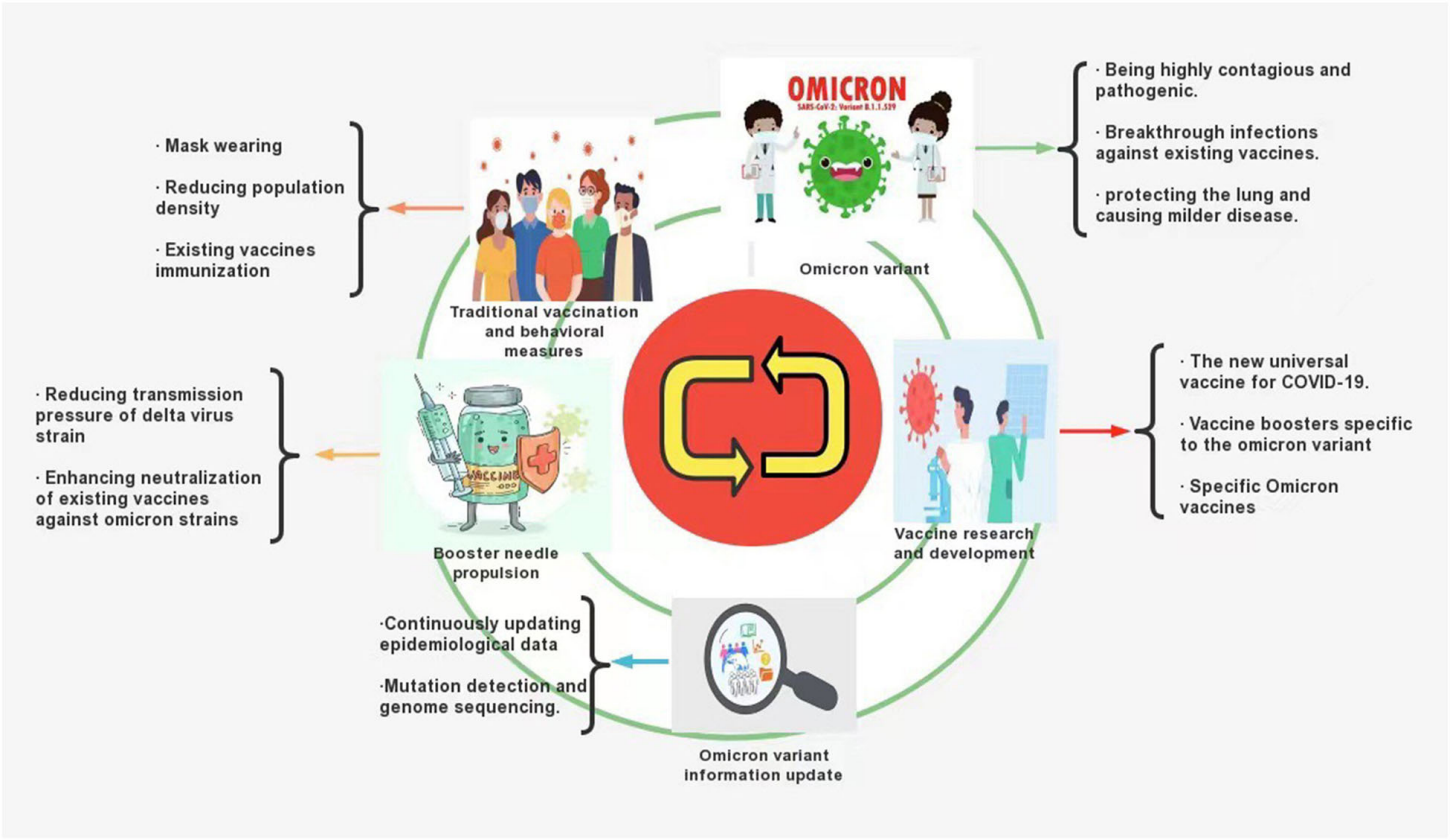
Strategies to mitigate COVID‐19 now and in the future^36^, ^37^, ^38^, ^44^, ^45^, ^46^, ^47^, ^48^

In conclusion, current proven interventions such as behavioral changes, vaccines, and booster shots should still be recommended and implemented.^46^ In addition, the characteristics of Omicron variant strains need to be summarized and confirmed based on future clinical data. Epidemic prevention measures must be adjusted, according to the target, to defend against the double attack of the Delta and Omicron strains, while we wait for new vaccines and optimized vaccination programs in the future (Figure 4). By being prepared for the Omicron variant, we will be better prepared for the next wave, for better or worse.

## CONFLICTS OF INTEREST

The authors declare no conflicts of interest.

## AUTHOR CONTRIBUTIONS

*Visualization, writing—original draft preparation, writing—review and editing*: Kaixi Ding. *Writing—original draft preparation, writing—review and editing*: Wei Jiang. *Writing—review and editing*: Chunping Xiong. *Conceptualization, project administration, writing—review and editing*: Ming Lei.

## Supporting information

Supplementary information.

## Data Availability

Data sharing is not applicable to this article as no new data were created or analyzed in this study.
