# Supplementary material for: Turning point: A new global COVID‐19 wave or a signal of the beginning of the end of the global COVID‐19 pandemic?
Source: Immun Inflamm Dis. 2022 Mar 29;10(4):e606. doi: 10.1002/iid3.606 (PMC8962637; doi:10.1002/iid3.606)
Supplement: Supplementary file 1 — Supplementary information. [file IID3-10-0-s001.pdf]

Home | Help | Live Chat | jerry ding

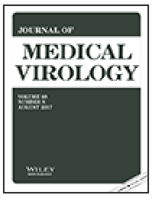

Characterization of the novel SARS-CoV-2 Omicron (B.1.1.529) variant of concern and its global perspective

Author: Shailendra K. Saxena, Swatantra Kumar, Saniya Ansari, et al

Publication: Journal of Medical Virology

Publisher: John Wiley and Sons

Date: Jan 11, 2022

© 2022 Wiley Periodicals LLC

Order Completed

Thank you for your order.

This Agreement between Mr. jerry ding ("You") and John Wiley and Sons ("John Wiley and Sons") consists of your license details and the terms and conditions provided by John Wiley and Sons and Copyright Clearance Center.

Your confirmation email will contain your order number for future reference.

License Number	5240780143039
License date	Feb 02, 2022

Printable Details

Licensed Content

Licensed Content Publisher	John Wiley and Sons
Licensed Content Publication	Journal of Medical Virology
Licensed Content Title	Characterization of the novel SARS-CoV-2 Omicron (B.1.1.529) variant of concern and its global perspective
Licensed Content Author	Shailendra K. Saxena, Swatantra Kumar, Saniya Ansari, et al
Licensed Content Date	Jan 11, 2022
Licensed Content Volume	0
Licensed Content Issue	0
Licensed Content Pages	7

Order Details

Type of use	Journal/Magazine
Requestor type	University/Academic
Is the reuse sponsored by or associated with a pharmaceutical or medical products company?	no
Format	Print and electronic
Portion	Figure/table
Number of figures/tables	2
Will you be translating?	Yes, including English rights
Number of languages	1
Circulation	1 - 29

About Your Work

Title of new article	Turning point: a new global COVID-19 wave or a signal of the beginning of the end of the global COVID-19 pandemic?
Lead author	Kaixi Ding
Title of targeted journal	Immunity, Inflammation and Disease
Publisher	John Wiley and Sons Ltd
Expected publication date	Feb 2022

Additional Data

Order reference number	6
Portions	Figure 1, on page 4.
Specific Languages	English

Requestor Location

	Mr. jerry ding<br>sichuan province chengducity<br>china
Requestor Location	chengdu, 86<br>China<br>Attn: Mr. jerry ding

Tax Details

Publisher Tax ID	EU826007151

Price

Total	0.00 USD

Would you like to purchase the full text of this article? If so, please continue on to the content ordering system located here: Purchase PDF
If you click on the buttons below or close this window, you will not be able to return to the content ordering system.

Total: 0.00 USD

CLOSE WINDOW ORDER MORE

© 2022 Copyright - All Rights Reserved | Copyright Clearance Center, Inc. | Privacy statement | Terms and Conditions
Comments? We would like to hear from you. E-mail us at customercare@copyright.com
